# Effects of Walking as Physical Exercise on Functional Limitation through Pain in Patients with Fibromyalgia—How Does Catastrophic Thinking Contribute?

**DOI:** 10.3390/ijerph20010190

**Published:** 2022-12-23

**Authors:** Patricia Catalá, Cecilia Peñacoba, Sofía López-Roig, María Angeles Pastor-Mira

**Affiliations:** 1 Department of Psychology, Rey Juan Carlos University, Alcorcón, 28922 Madrid, Spain; 2 Department of Behavioral Sciences and Health, Miguel Hernandez University, Elche, 03550 Alicante, Spain

**Keywords:** fibromyalgia, walking, pain, catastrophizing, functional limitation, moderate mediation

## Abstract

Background: Aerobic exercise has a beneficial impact on physical and mental health. However, patients with fibromyalgia do not always report perceiving these improvements. Objective: The aim of this study was to examine whether catastrophic thinking moderated the effects of perceived pain severity once an active and regular lifestyle had been established on functional limitation in chronic pain patients, in particular in fibromyalgia patients. Methods: The sample consisted of a total of 491 women with fibromyalgia diagnosed according to the criteria of the American College of Rheumatology. Participants completed an ad-hoc item about lifestyle related to walking pattern, the Brief Pain Inventory, the Pain Catastrophization Scale, and the Fibromyalgia Impact Questionnaire-Revised. To examine the relationship between the variables, a moderate mediation analysis was performed through the macro PROCESS (model 14). Results: The relationship between the performance of the recommended walking pattern and functional limitation was mediated by the severity of pain (B = −5.19, SE = 1.59, t = −3.25, 95% CI = [−4.06, −0.28], *p* < 0.001). Furthermore, it was found that the mediating effect of pain severity was moderated by catastrophic thinking (Index = −0.014, SE = 0.007, 95% CI [0.002, 0.030]). Conclusions: The positive effect of walking on functionality through the reduction of pain levels is favored when patients present low catastrophizing, which affects the relevance of including interventions focused on the reduction of catastrophizing in the prescription of physical exercise in patients with fibromyalgia as the treatment of choice.

## 1. Introduction

Aerobic exercise is considered as one of the most effective primary treatments to improve the well-being of patients with fibromyalgia [1,2]. Within the different modalities of aerobic physical exercise, walking offers greater advantages, such as self-regulation of the pattern itself, low economic cost, and minimal interference in normal routines, and relies exclusively on the motivation of the patient [3]. The walking behavior proposed by Gusi et al. [4] for fibromyalgia patients was “walking at least 60 min in bouts of 20 min, with a small rest between bouts, four times a week, over a minimum of six consecutive weeks”. Compliance with this long-term guideline appears to have important repercussions on different health-disease outcomes of these patients [5]. Reducing physical fatigue, disability, the impact of the disease on daily life, or improving pain management are some of the most prominent benefits [4,6,7]. However, not all patients with fibromyalgia report perceiving improvements in their physical health after performing the activity [8,9,10,11]. This discrepancy could be due to the severity of pain experienced by the patients [12], which has been shown to predict functional limitation in a previous study [13]. Taking into account that generalized chronic pain is the central symptom of fibromyalgia [14,15] and that it leads to changes in the individual’s ability to function physically [13,16], it is possible that this mediates the relationship between walking behavior, improvements in functional limitation, and the impact of the disease in general.

Furthermore, the effect of pain severity on functionality could be accelerated by the patients’ cognitive processing. Cognitive processing includes various mental operations carried out by the brain to process information (i.e., thoughts) and allow behavior to be modulated in the face of certain stimuli such as the presence of pain [17]. Previous literature agrees that cognitive processing contributes directly to individual differences regarding functional limitation [18,19].

At a clinical level, one of the most significant cognitive processes, with a greater presence in patients with fibromyalgia, is catastrophic thinking [18,20]. Castastrophization is defined as an exaggerated or increased negative “mentality” about anticipated or real situations associated with a certain event, such as experiencing pain [21]. From an affective-motivational perspective, people with chronic pain must manage multiple goals that may be incompatible with each other, such as pain avoidance and being active. Therefore, the presence of pain becomes an inconvenience in achieving other important goals for women with fibromyalgia. Thus, catastrophic thinking would be a reflection of concern about that interference [22]. Furthermore, the fear-avoidance model of pain predicts that perceiving pain as a catastrophe increases symptoms, emotional suffering, or disability [12,23,24,25]. The most current literature adds that catastrophic thinking would act as a moderator in the association between activity patterns and the impact of the disease [26]. In contrast, there are no studies that have evaluated whether catastrophic thinking affects the relationship between pain severity and functional limitation in fibromyalgia patients. Within a more global model, the objective of this manuscript has been to examine: (1) the effects of walking regularly on functional limitation mediated by the role of pain perception, and to analyze (2) how the relationship between pain and functional limitation can be moderated by catastrophizing. Considering what is proposed by the previous literature, it is hypothesized that pain severity mediates (negatively) the relationship between walking behavior and functional limitation and that cognitive processes such as catastrophizing moderate (positively) the mediating effect.

## 2. Materials and Methods

### 2.1. Design

This was a cross-sectional study involving 491 women with fibromyalgia diagnosed according to the criteria of the American College of Rheumatology (ACR) [27]. The sample was selected for convenience by contacting several patient associations from different Spanish regions. In addition to the diagnosis of fibromyalgia, our inclusion criteria were: being a female (for homogeneity purposes, because FM patients are mostly females), being over 18 years of age, having medical advice to walk, and having the physical and mental ability to provide informed consent and to complete the surveys. Exclusion criteria were to have physical comorbidity or any other pathology that prevented carrying out the walking pattern and not signing the informed consent form. Once the participants gave their informed consent to participate in the project, they were given a booklet of questionnaires that took between 20 and 30 min to complete. This multicenter study was conducted according to the guidelines of the Declaration of Helsinki and approved by the Institutional Review Board (or Ethics Committee) of Universidad Miguel Hernández (Reference DPS.MPM.02.16, date of approval 20 December 2016), Hospital General de Alicante (Reference PSI2016-79566-C2-1-R, date of approval 30 November 2016), and Universidad Rey Juan Carlos (Reference 160520165916, date of approval 9 June 2016).

### 2.2. Instruments

Walking pattern (regular walking): This was based on the one established by Gusi et al. [4], however, the minimum daily time was reduced to 30 min and at least 2 days a week because the targeted population was highly sedentary and because of the difficulties in adherence to physical exercise in patients with fibromyalgia [1,28]. An ad hoc item was used to assess whether they adhered to walking according to the prescribed pattern, specifically, “walking at least 30 min in bouts of 15 min, with a small rest between bouts, twice a week, over a minimum of six consecutive weeks”. A dichotomous response format was used (1 = “yes” or 0 = “no”).

Severity of pain: To assess pain severity, we used the mean score of the four pain severity items from the Brief Pain Inventory [29]: maximum, minimum, and general intensity of pain during the last 7 days and intensity of pain in the present moment. Each rating was assessed using an 11-point numerical scale ranging from 0 (no pain) to 10 (worst pain imaginable). This procedure for measuring pain intensity has been widely used in the pain literature [30]. In this study, the internal consistency for this scale was high (α = 0.86).

Pain catastrophizing: The global score of the Spanish adaptation of the Pain Catastrophization Scale (PCS) was used for this study [31]. This scale is made up of 13 items that are evaluated using a 5-point Likert-type response scale ranging from 0 (not at all) to 4 (always). The scale contains items like “I worry all the time about whether the pain will end” or “I feel can’t go on”. High scores in this dimension indicate a greater degree of catastrophism. The internal consistency of this scale for this study was 0.87.

Functional limitation: The physical functionality dimension of the Spanish adaptation of the Fibromyalgia Impact Questionnaire-Revised (FIQ-R) [32] was used in this study. This dimension is made up of 10 items with several sub-elements (a–j), each with a range of 4 points (from 0, always, to 3, never) scored on a Likert-type scale. These items assess the functional capacity of the patient through questions about activities related to daily life: shopping, driving, doing laundry, etc. High scores indicate greater functional limitation. The Cronbach’s alpha for functional limitation in this study was 0.85.

Anxiety and Depression: The Spanish version of the Hospital Anxiety and Depression Scale (HADS) [33] was used. This instrument has been widely used to measure the possible presence of states of anxiety and depression in medical, non-psychiatric, outpatient clinic settings. The dimensions of anxiety and depression are composed of 7 items each with a 4-point Likert-type response format. High scores on these dimensions indicate higher levels of the symptom. Cronbach’s alpha in this study was 0.85 for the anxiety dimension and 0.82 for the depression dimension.

Sociodemographic and clinical data: To assess age, educational level, employment status and marital status an ad-hoc questionnaire was used. Regarding the clinical variables, the duration of fibromyalgia and specialty where patient was diagnosed were evaluated.

### 2.3. Data Analysis

**Figure 1:**
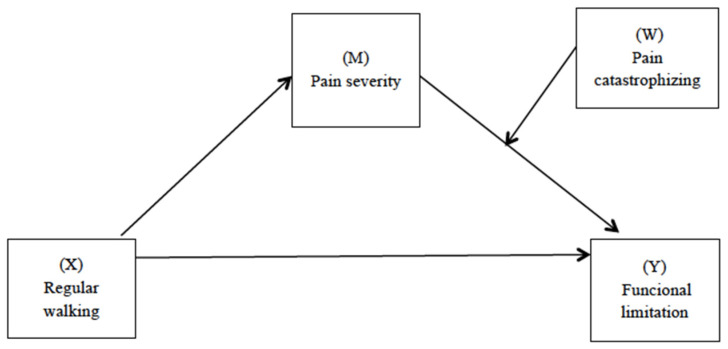
Path diagram illustrating the moderate mediation model.

Data were analyzed using IBM SPSS statistics 22.0 software [34] and PROCESS macro v3.3 for SPSS [35]. Descriptive statistics (percentages, mean with standard deviation and observed score range) were used to evaluate the characteristics of the sample and the distribution of the variables under study. The correlations between the main variables were examined using Pearson’s correlation coefficients. A simple mediation analysis (model 4) proposed by Hayes (2017) was performed to examine the mediating effects of pain severity (M) in the association between walking regularly (X) and functional limitation (Y). Finally, moderate mediation analyzes (model 14) proposed by Hayes (2017) were performed to investigate whether catastrophism (W) moderates the indirect effect of walking (X) on functional limitation (Y) through pain (M) (Figure 1). In order to include these variables in the models, it was previously verified that they correlated with each other. For both models, 5000 bootstrap samples and a 95% confidence interval were selected. Statistical significance was defined as a two-tailed *p*-value of <0.01. To assess indirect conditional effects of walking behavior on functional limitation through pain severity, depending on different levels of catastrophizing, the bootstrap method was used. The 95% confidence interval (CI) from this method were used to examine indirect effects at three levels of catastrophism (1 standard deviation (SD) above the mean, at the mean, and 1 SD below the mean). In order to control for the effect of relevant variables in the model, age, depression, and anxiety were included as covariates.

## 3. Results

### 3.1. Sample Characteristics

The mean age of the sample was 53.89 years (SD = 9.25), which ranged from 19 to 78 years. Seventy-five percent of the women were married or in a stable relationship, 12% were separated or divorced, 8% were single, and 5% were widows. Regarding educational level, 24% of the women had university studies, 46.3% had secondary studies, 27% had primary studies, and 2.7% could read and write. Regarding the clinical variables, the patients had been diagnosed with fibromyalgia for a mean of 9.85 years (SD = 8.49; range 1–46 years). Most of the sample had been diagnosed in rheumatology units (70.6%, n = 331), only 10.2% (n = 48) had been diagnosed in primary care units, and a scarce 3% (n = 14) in trauma units. The rest of the participants (16.2%, n = 76) had received their diagnoses in rehabilitation, neurology, or pain and fibromyalgia units, among others.

### 3.2. Descriptive Analysis and Correlations

Table 1 shows the characteristics and correlates of pain severity, functional limitation, catastrophizing, walking behavior, anxiety, and depression. Walking behavior was negatively correlated with pain intensity, catastrophizing, functional limitation, and anxiety (*p* < 0.001). Functional limitation was positively correlated with pain intensity, pain catastrophism, anxiety, and depression (*p* < 0.001). Pain intensity was positively correlated with catastrophizing, anxiety, and depression (*p* < 0.05). Likewise, catastrophizing was related to anxiety and depression (*p* < 0.05). There were no significant differences in functional limitation scores with regard to sociodemographic and clinical variables (all *p* > 0.05) except for age (*p* = 0.034).

### 3.3. Mediation Model of the Relationship between Regular Walking and Functional Limitation with Pain Intensity as a Mediator

**Figure 2:**
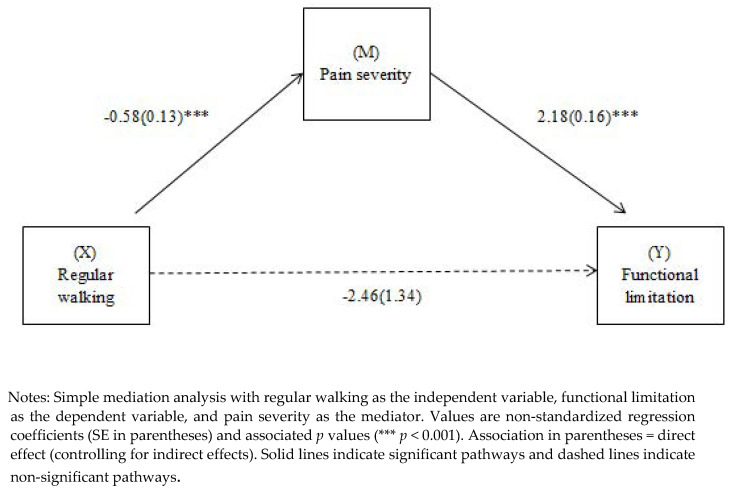
Path diagram illustrating direct and mediating effects that relate regular walking to functional limitation (with pain severity as mediator).

The results of the mediation analysis are shown in Figure 2. Depression, anxiety, and age were used as covariates in the model. The total effect model was significant (B = −5.19, SE = 1.59, t = −3.25, 95% CI = [−4.06, −0.28], *p* < 0.001). The effect of regular walking on functional limitation was completely mediated by pain intensity while controlling for demographic and clinical variables. There was no direct effect of regular walking on functional limitation. The total amount of variance explained by the model was 41% (F = 68.65, *p* < 0.001).

### 3.4. Moderate Mediation Model

Table 2 shows the moderate mediation analyses that include pain severity as a mediator when catastrophizing is used as a moderator in the relationship between pain severity and functional limitation. Anxiety, depression, and age were used as covariates in the model. The results showed that the contribution of pain on functional limitation varied at different values of catastrophizing in patients who walked regularly after controlling for covariates (Index = −0.014, SE = 0.007, 95% CI [0.002, 0.030]). As shown in Table 3, the mediating effect of pain severity varied according to the intensity of pain catastrophism, being more intense 1 SD below the mean (B = −1.22). This indicates that the positive effect of walking on limitation through the decrease in pain levels is favored when patients present low catastrophism. The evaluated model explains 59% of the variance of the functional limitation. No multicollinearity problems were found in the analyses (tolerance values greater than 0.010).

## 4. Discussion

The present study analyzed the association between having an active lifestyle (i.e., regular walking pattern), pain severity, catastrophic thinking, and functional limitation through a moderate mediation analysis. Specifically, the intention was to explore whether carrying out an active lifestyle whilst adapting (by reducing the level of demand) the walking guideline recommended by Gusi et al. [4] for fibromyalgia patients positively influenced physical functionality through pain, and whether pain catastrophizing moderated the effect of pain on disability. The results obtained showed that the relationship between regular walking and functional limitation was mediated by the severity of pain. Furthermore, the effects were found to be especially significant at low levels of catastrophizing.

In line with the first objective of this study, we found two main findings. First, the effect of walking on functional limitation was mediated by pain severity. Specifically, it seems that leading an active lifestyle predicts less intensity of pain and this, in turn, less functional limitation. Second, and unlike what has been found in previous literature [36,37], the results did not show a direct relationship between walking behavior and functional limitation, i.e., walking regularly only influences the perception of improved functionality if the patients have previously perceived a decrease in pain levels. This finding is consistent with previous research pointing to the important benefits of aerobic exercise on the health status of patients with chronic pain [1,2]. Likewise, these results coincide with the hypothesis proposed by Terrier et al. [5], who suggest that it is necessary to lead a structured and prolonged active lifestyle so that patients with fibromyalgia can perceive a decrease in the levels of pain. In addition, the results obtained in our study incorporate and suggest that this fact has a positive impact on the daily functioning of these patients [4,6,7].

Likewise, when performing a moderate moderation model, the results confirmed that catastrophizing levels play a moderating role in the influence of pain on functional limitation in patients who walk regularly. Specifically, low levels of catastrophism allow us to perceive an improvement in functional limitation despite pain. These results are in agreement with Ellingson et al. [38], who showed how catastrophizing interferes with the ability of people with fibromyalgia to distract themselves from pain. This could explain the modulatory nature of catastrophism in the mediating effect of pain in the relationship between regular walking and functional limitation. Following this evidence, a novel aspect of this research is that it indicates one of the mechanisms of action of the improvement that regular walking produces in functional limitation despite pain. It is striking that this effect is especially relevant at low levels of catastrophism. To date, most research has emphasized the negative consequences of presenting high levels of catastrophism [39,40,41], leaving aside the effect that low levels of this variable could have. In our opinion, these results have important clinical repercussions since patients with fibromyalgia could have better results in their functionality if they perceived less pain after walking and if they used cognitive resources appropriately. Transferring these findings to a clinical context, it is suggested that health professionals work so that patients are able to adhere to the walking pattern until they perceive a decrease in pain levels, since it is known that in the first days or weeks pain levels may increase after activity [42]. This finding highlights the need to develop multicomponent intervention programs where symptoms are addressed from different healthcare disciplines. Establishing programs that contain, in addition to pharmacological treatment, specific physical and psychological techniques (that is, walking following the appropriate guidelines for chronic pain and carrying out interventions based on managing thoughts) could improve the quality of life of patients with fibromyalgia.

Finally, a series of limitations must be considered to generalize the results. In the first place, carrying out a cross-sectional design does not allow to infer cause-effect relationships between walking behavior, pain severity, pain catastrophizing, and functional limitation, so longitudinal studies are needed. Second, the sample consisted only of female patients with fibromyalgia, which does not guarantee the generalizability of the findings to other populations with chronic pain or the male population with this disease. It would be convenient, therefore, to carry out studies of this type in different populations to corroborate what is stated here. Third, the evaluations were carried out through self-report questionnaires, which could lead to a bias in the responses and affect the results. In future research, carrying out a follow-up through mobile applications or accelerometers to verify compliance with the walking guideline and include semi-structured interviews could provide more accurate and truthful information on the lifestyle and general health of patients. However, this problem is common in the previous existing literature in this context [43]. The debate over whether objective or subjective measures of pain and related outcomes should be used in people with chronic pain has been a concern for decades, and multimodal measurement, i.e., a combination of both, appears to be preferable when possible [44]. Finally, it is important to point out that the study only included catastrophism as the main cognitive process given the relevance marked by the previous literature on fibromyalgia [12,23,24,25]. However, this list is far from complete and other psychosocial factors, such as cognitive fusion, acceptance, or motivation, could also be taken into account in future research.

## 5. Conclusions

Despite the aforementioned limitations, we can conclude that this study presents relevant results both for the clinical setting and for the field of current research. Specifically, we found that adhering to the walking pattern was significantly associated with functionality through pain. In addition, the positive effect of walking on limitation, through the decrease in pain levels, is favored when patients have low levels of catastrophization.

## Figures and Tables

**Table 1:** Descriptives and correlations between variables (n = 491).

Psychosocial Characteristics	Mean (SD)	Sample Range	2.	3.	4.	5.	6.	7.
1. Pain severity	7.05 (1.49)	1–10	0.367 **	0.541 **	0.219 **	0.172 **	0.058	−0.187 **
2. Pain catastrophizing	30.20 (12.17)	0–52		0.401 *	0.497 **	0.188 **	−0.052	−0.191 **
3. Functional limitation	22.00 (6.27)	0–30			0.255 **	0.212 **	0.096 *	−0.222 **
4. Axiety	12.17 (3.69)	4–21				0.113 *	−0.107 *	−0.159 **
5. Depression	11.48 (4.15)	1–21					0.107	−0.073
6. Age	53.89 (9.25)	19–78						−0.062
7. Regular walking, n (%)								
Yes	312 (63.5)			.	.			
No	179 (36.5)							

* *p* < 0.05, ** *p* < 0.01; Abbreviations: SD (standard deviation); n (number); % (percentage).

**Table 2:** Moderate mediation analysis assuming pain catastrophizing as mediator (outcome variable = functional limitation).

	*R* ^2^	*F*	*p*	Beta	t	*p*
Model 1	0.59	66.91	<0.001			
Regular walking				−2.46	−1.83	0.067
Pain severity				2.54	7.10	<0.001
Pain catastrophizing				0.28	3.36	<0.001
Pain catastrophizing × Pain severity				−0.02	−2.10	0.033
Anxiety (covariate)				0.079	1.10	0.271
Depression (covariate)				0.130	2.29	0.021
Age (covariate)				0.032	1.29	0.195

**Table 3:** Indirect conditional effect at specific levels of the moderator when treating pain severity as a mediator.

Pain Catastrophizing	Beta	SE	LL 95% CI	UL 95% CI
1SD below the mean	−1.22	0.31	−1.86	−0.64
Mean	−1.05	0.27	−1.61	−0.54
1SD above the mean	−0.87	0.25	−1.41	−0.42

Notes: SE = standard error; LL 95% CI = lower level of the 95% confidence interval; UL 95% CI = upper level of the 95% confidence interval.

## Data Availability

The data presented in this study are available on request from the corresponding author. The data are not publicly available due to privacy restrictions.
